# Antibiofilm effects of *N*,*O*-acetals derived from 2-amino-1,4-naphthoquinone are associated with downregulation of important global virulence regulators in methicillin-resistant *Staphylococcus aureus*

**DOI:** 10.1038/s41598-020-76372-z

**Published:** 2020-11-12

**Authors:** Juliana Silva Novais, Mariana Fernandes Carvalho, Mariana Severo Ramundo, Cristiana Ossaille Beltrame, Reinaldo Barros Geraldo, Alessandro Kappel Jordão, Vítor Francisco Ferreira, Helena Carla Castro, Agnes Marie Sá Figueiredo

**Affiliations:** 1 Programa de Pós-graduação em Ciências e Biotecnologia, Instituto de Biologia, Universidade Federal Fluminense, Campus Valonguinho, Niterói, RJ 24210-130 Brazil; 2 Universidade Federal do Rio de Janeiro, Instituto de Microbiologia Professor Paulo de Góes, Departamento de Microbiologia Médica, Rio de Janeiro, 21941902 Brazil; 3 Departamento de Farmácia, Centro de Ciências da Saúde, Universidade Federal do Rio Grande do Norte, Natal, 59012-570 Brazil; 4 Departamento de Química Orgânica, Instituto de Química, Universidade Federal Fluminense, Campus do Valonguinho, Niterói, 24020-007 Brazil

**Keywords:** Drug discovery, Microbiology

## Abstract

Despite the existing antibiotics, antimicrobial resistance is a major challenge. Consequently, the development of new drugs remains in great demand. Quinones is part of a broad group of molecules that present antibacterial activity besides other biological properties. The main purpose of this study was to evaluate the antibiofilm activities of synthetic *N*,*O*-acetals derived from 2-amino-1,4-naphthoquinone [**7a**: 2-(methoxymethyl)-amino-1,4-naphthoquinone; **7b**: 2-(ethoxymethyl)-amino-1,4-naphthoquinone; and **7c**: 2-(propynyloxymethyl)-amino-1,4-naphthoquinone] against methicillin-resistant *Staphylococcus aureus* (MRSA). The derivatives **7b** and **7c**, specially **7b**, caused strong impact on biofilm accumulation. This inhibition was linked to decreased expression of the genes *fnbA*, *spa*, *hla* and *psmα3*. More importantly, this downregulation was paralleled by the modulation of global virulence regulators. The substitution of 2-ethoxymethyl (**7b**) in comparison with 2-propynyloxymethyl (**7c**) enhanced *sarA*-*agr* inhibition, decreased *fnbA* transcripts (positively regulated by *sarA*) and strongly impaired biofilm accumulation. Indeed, **7b** triggered intensive autolysis and was able to eliminate vancomycin-persistent cells. Consequently, **7b** is a promising molecule displaying not only antimicrobial effects, but also antibiofilm and antipersistence activities. Therefore, **7b** is a good candidate for further studies involving the development of novel and more rational antimicrobials able to act in chronic and recalcitrant infections, associated with biofilm formation.

## Introduction

Despite being part of the human microbiota, *Staphylococcus aureus* can cause pathological conditions varying from skin/soft tissue infections to more severe diseases, such as pneumonia, bloodstream infections (BSI), osteomyelitis, and endocarditis. These bacteria are a major cause of both hospital-associated and community-acquired infections worldwide^1^. The *S. aureus* genomic plasticity has facilitated its accelerated evolution as the consequence of the lateral acquisition of new traits, including antimicrobial resistance genes^2^.

The first case of methicillin-resistant *S. aureus* (MRSA) was reported in 1961, in England^3^. After that, MRSA rapidly spread throughout the world, becoming one of the main hospital-associated pathogens^1^. The pathogenicity of *S. aureus* infections is multifactorial and directly related to the production of a plethora of virulence factors and their interactions with the host^4^. The development of biofilm by *S. aureus* is considered the most important mode of growth lifestyle for some infections, mainly those related to implantable medical devices such as catheters, and to cardiac and orthopedic prostheses^5^. Also, biofilms can have other negative impacts on bacterial infections because bacterial cells in the biofilm growth do not efficiently respond to either antimicrobials or the killing promoted by the immune system^5–7^.

Some hypotheses have been proposed to explain the antimicrobial refractory/persistence in biofilms: (1) impairment of the antimicrobial penetration in the extracellular matrix; (2) activation of efflux pumps; (3) activation of stringent response with generation of persister cells due to increased concentration of the penta- or tetra-phosphate alarmone, commonly referred as (p)ppGpp; (4) specific genetic background of the bacterium; and (5) stochastic phenomenon, where a small population acquires a specific gene expression profile that leads to antimicrobial persistence^5,8^. The occurrence of subpopulations of bacteria that display antimicrobial persistence reinforces the importance of the discovery of new molecules that can kill persistent cells. However, the development of new antibacterial agents has decreased in recent decades^9,10^. In previous studies, using broth macrodilution for MIC determination, we have shown that some *N*,*O*-acetal 2-amino-1,4-naphthoquinone derivatives presented promising action against planktonic Gram-positive bacteria^11^. In the study presented here, we demonstrated that 2-ethoxymethyl 2-amino-1,4-naphthoquinone, besides presenting acceptable pharmacological and toxicological parameters through in silico and ex-vivo studies, also displays other desirable properties associated with antibiofilm and antipersistence effects. In addition, we investigated the mechanisms involved in the modulation of biofilm accumulation by this compound.

## Materials and methods

### Bacterial strains and growth conditions

Strain representatives of six globally spread clones/lineages of MRSA were used in this study to test the antimicrobial activity of the *N*,*O*-acetal derivatives against MRSA: NY/Japan-USA100/ST5-SCC*mec*II (strain CR15-071), USA300/ST8-SCC*mec*IV (strain USA300-0114), USA400/ST1-SCC*mec*IV (strain USA400-0051), Brazilian epidemic clone-BEC/ST239-SCC*mec*III (strain BMB9393), pediatric clone-USA800/ST5-SCC*mec*IV (strain USA800-0179), and OSPC-USA1100/ST30-SCC*mec*IV (strain 07-040). The *S. aureus* ATCC 25923 was used as a control for the experiment of drug susceptibility. The biofilm-producing strain BMB9393 (ST239-SCC*mec*III) was chosen for biofilm assays because its superior capability to accumulate biofilm matrix compared with other MRSA strains^12^. For biofilm production, tryptic soy broth (TSB; BD, Franklin Lakes, NJ, USA) was supplemented with 1% (w/v) glucose (TSB-Glu). Tryptic soy agar (TSA; BD) was used for bacterial growth. The Mueller-Hinton broth (MHB; BD) and Mueller-Hinton agar (MHA; BD) was used for susceptibility tests. All strains were maintained in 10% (v/v) glycerol stocks and stored at − 80 °C.

### *N*,*O*-acetal derivatives

To obtain *N*,*O*-acetals derivatives from 2-amino-1,4-naphthoquinone (**6a**), a methodology that employs microwave irradiation was used as described previously^11^. The **6a** and its derivatives **7a** [2-(methoxymethyl)-amino-1,4-naphthoquinone], **7b** [2-(ethoxymethyl)-amino-1,4-naphthoquinone] and **7c** [2-(propynyloxymethyl)-amino-1,4-naphthoquinone] were characterized using spectroscopic methods, such as ^1^H and ^13^C NMR^11^. Stock solutions of these derivatives were prepared in DMSO 100%. In the experiments, the final concentrations of DMSO were ≤ 2% (v/v), which showed in previous tests performed in this study to cause no effect in *S*. *aureus* planktonic or sessile growth.

### Susceptibility tests

The antibacterial activity of **6a** and its derivatives **7a**, **7b**, and **7c** was tested as previously described using paper disks impregnated with each compound^13^. The derivatives were also analyzed quantitatively using microdilution assays in 96-well inert polystyrene microtiter plates (Nuclon; Nalge Nunc International, Rochester, NY, USA) to determine the minimal inhibitory concentration (MIC)^14^. Biological control, as well as growth conditions, were performed following CLSI recommendation^15,16^. Additionally, the MIC values of the MRSA strains BMB9393 (BEC) and CR15-071 (USA100), and the control strain ATCC25923, for the derivatives **7a**, **7b** and **7c** were also determined using Mueller Hinton agar dilution following CLSI recommendation^16^. Controls were also performed using 2% (v/v) DMSO only.

### Cytotoxicity assay

The Vero cell line was cultivated to form monolayers on 96-well flat-bottom culture plates with Dulbecco’s modified Eagle’s medium (DMEM; Thermo Fisher Scientific, Waltham, MA, USA ) supplemented with 5% (v/v) fetal bovine serum (FBS; Thermo Fisher Scientific), 1 µg/mL fungizone, 2.5 Mm l-glutamine (amino acid), and 100 U/mL penicillin, during 24-h incubation period at 37 °C and 5% carbon dioxide (CO_2_). After that, the supernatant was removed, and the cell monolayers were incubated for 24 h with different concentrations of **6a** or its derivatives **7a**, **7b**, or **7c**, ranging from 8 to 512 µg/mL. For comparative purposes, these experiments were also performed with the commercially available antibiotics, tetracycline and trimethoprim-sulfamethoxazole, using the same range of concentrations. All compounds were diluted in DMSO for a final concentration of 1% (v/v) in the cell monolayers. In the experimental controls, DMSO was added to the drug-untreated cell monolayers for a final concentration of 1% (v/v). After incubation, the supernatant was removed, and 100 µL fresh medium and 20 µL 3-[4,5-dimethylthiazole-2-y]-2,5-diphenyltetrazolium bromide (MTT; 1 mg/mL) were added into each well. Plates were incubated for 1 h, the supernatant again removed, and 200 µL dimethyl sulfoxide (DMSO) were added to each well to dissolve the formazan crystals for 10 min. Absorbance was measured at 570 nm using an ELISA reader (Thermo Plate, TP-READER, Thermo Fisher Scientific). The cytotoxic concentration 50% (CC_50_) value was defined as the concentration of a derivative required for reducing cell viability by 50%. The selectivity index (SI) was calculated as follows: SI (µg/mL) = CC_50_/MIC^17^. Three independent experiments were performed in triplicate. Controls were performed using 2% (v/v) DMSO only.

### Hemocompatibility assay

2-Amino-1,4-naphthoquinone and its derivatives **7a**, **7b**, and **7c** were also evaluated for their hemolytic activity, using final concentrations of 25 µg/mL, 50 µg/mL, 100 µg/mL and 300 µg/mL in 1% (v/v) DMSO, according to Sathler and collaborators^18^. Erythrocytes were collected from three human volunteers in a citrate tube and washed three times (3×) with PBS (pH 7.4). All derivatives were incubated with the PBS-erythrocyte suspension for 3 h at 37 °C. The hemolysis with the release of hemoglobin was quantified by the spectrophotometric reading of the supernatant at 545 nm. Hemolysis less than 10% represented hemocompatibility and non-toxicity against erythrocyte membranes^19^. For the positive control, the human erythrocytes were lysed with 1% (v/v) Triton X-100. The negative control was performed with 1% (v/v) DMSO only (final concentration). This protocol was approved by the Human Research Ethics Committee of the Universidade Federal Fluminense and under the number #20870414.9.0000.5243. All methods were carried out in accordance with relevant guidelines and regulations. In addition, informed consent was obtained from all volunteers. Three independent experiments were performed in triplicate.

### ADMET properties

The in silico pharmacokinetic properties and toxicity profiles (ADMET) of **6a** and its derivatives **7a**, **7b** and **7c** were evaluated using pkCSM—pharmacokinetic webserver (https://biosig.unimelb.edu.au/pkcsm/)^20^, Pro-Tox-II webserver (https://tox.charite.de /protox_II/)^21^ and SwissADME webserver^22^. These results were also compared with the profiles of an antimalarial (atovaquone) and six antimicrobials (cefoxitin, ciprofloxacin, furazolidone, nitrofurantoin, tetracycline, and vancomycin). The theoretical pharmacokinetic properties evaluated were absorption, distribution, metabolism, and excretion (ADME). The absorption parameters were tested through simulation modelling of human intestinal absorption using Caco-2 permeability, human intestinal absorption, P-glycoprotein substrate, and P-glycoprotein I/II inhibition. For drug distribution, it was analyzed the steady-state volume of distribution (Vss) and the blood–brain barrier penetration (BBBP) predictions. Drug metabolism was analyzed by the theoretical activity of cytochrome P450 (CYP) enzymes. Lastly, excretion parameters including theoretical total clearance and renal OCT2 substrate were evaluated. Toxicological analyses comprised toxicity target, hepatotoxicity, hERGI/II inhibitors, and toxicity endpoints including carcinogenic, mutagenic, immunotoxicity, and cytotoxicity parameters.

### Effect of *N*,*O*-acetal derivatives on biofilm development

Biofilms were formed on polystyrene surfaces using the BEC representative, strain BMB9393. It had previously been shown that this method has a good correlation with an in vivo biofilm model^23^. The compound **6a** (1MIC = 128 µg/mL) and its derivatives **7a** (1MIC = 256 µg/mL), **7b** (1MIC = 128 µg/mL), and **7c** (1MIC = 64 µg/mL) were added, separately, into each well of a polystyrene microtiter plate (Nunc) at concentrations of 1/4MIC 1/8MIC, or 1/16MIC in trypticase soy broth with 1% glucose (TSB-Glu; 2× final concentration). The same volume of the bacterial culture in TSB-Glu (37 °C/18 h; at 250 rpm) diluted 1/100 in the same broth was added to the wells. After incubation (37 °C/24 h), supernatants were gently removed, rinsed with distilled water, and treated with crystal violet solution as described^23^. The adhered biomass was suspended in ethanol PA, and the OD read at 570 nm using the SpectraMax Plus 384 (Absorbance Microplate; Molecular Devices, San Jose, CA, USA). Eight independent tests with three replicates each were performed. Controls were inoculated with the same bacterial inoculum but using only TSB-Glu with 2% (v/v) DMSO, final concentration.

### Confocal laser scanning microscopy (CLSM)

Because derivative **7b** showed the stronger reduction in biofilm accumulation, this compound was elected for these and other experiments designed to unveil the mechanisms by which this molecule may affect biofilm development. For comparative purposes and validation of the experiments performed here, the derivative **7c**, which exhibited a lesser but still important reduction on biofilm accumulation, was also included in these studies. For CLSM experiments, after 24 h of incubation, the supernatant containing planktonic cells was gently removed, and biofilm treated with 25 nM SYTO 9 DNA-intercalating stain (Invitrogen; Carlsbad, CA, EUA), as previously described^24^. Before visualization, SYTO 9 solution was removed and the material visualized using a confocal laser scanning microscope (Model LSM510 Carl Zeiss Meditec; Jena, Germany). The microscope was configured with the argon laser (458 nm/477 nm/488 nm/514 nm). The images were randomly captured with the Neorlu-Plan 406/06 Korr. Controls were performed using 2% (v/v) DMSO only (final concentration). Three independent experiments were performed in quadruplicate.

### Expression of biofilm-associated genes

Total RNA was obtained using the RNeasy Kit (Qiagen; Hilden, North Rhine-Westphalia, Germany) from sessile cells treated with 1/4MIC of the *N*,*O* acetal derivatives **7b** (32 µg/mL) or **7c** (16 µg/mL). Gene expression analyses were performed using real-time RT-qPCR with Power SYBR Green RNA-to-CTTM 1-Step Kit (Applied Biosystems, Foster City, CA, USA). The platform used was a One Step Real-Time PCR System (Applied Biosystems). The genes chosen were those well characterized as associated with biofilm development, including *fnbA*, *spa*, *mecA*, *hla*, and *psmα3*—encoding fibronectin-binding proteins A (FnBPA), staphylococcal protein A (Spa), penicillin-binding protein 2a or 2′ (PBP 2a or 2′), α hemolysin, and phenol-soluble modulin α3, respectively. To confirm the data from real-time RT-qPCR for the biofilm-associated genes, and to further understand the mechanisms by which **7b** caused a profound effect on MRSA biofilm, the same experiments were performed with the derivative **7c**. Additionally, these data were also validated by testing important biofilm and global virulence regulators, involved in the modulation of the biofilm-associated genes, such as RNAIII (the main effector molecule of the Agr quorum sensing), *sarA* (a global transcriptional regulator), and *sigB* (the alternative σ-factor B of the *S. aureus* RNA polymerase), using new RNA preparations. The validated PCR primers used for each gene tested are listed in the Supplementary Table S1. Relative quantification (RQ) of the target transcript was determined by calculating the comparative ΔΔCT using the 16S rRNA as the reference gene. Controls were performed using only the compound diluent, 2% (v/v) DMSO (final concentration). For each set of tests, three independent experiments were performed in triplicate.

### Effect of **7b** and **7c** derivatives in *S. aureus* autolytic system

The autolytic activity in the presence and absence of the derivatives **7b** or **7c** was measured by quantifying *atlA* cDNA by real-time qRT-PCR, as described above. To validate the RT-qPCR data for derivative **7b**, the same experiment was performed for derivative **7c**. Additionally, to confirm these data, quantification of extracellular DNA (eDNA) was performed in the supernatant of bacterial growth treated and untreated with 1/4MIC of these two derivatives (32 µg/mL and 16 µg/mL, respectively), using a previously described method^25^. Controls were performed using 2% (v/v) DMSO only. Three independent experiments were performed in triplicate.

### Effect *N*,*O*-acetal derivatives against subpopulations of MRSA cells persistent to vancomycin

For this test, we choose the MRSA strain CR15-071 representative of the ST5-SCC*mec*II lineage (NY/Japan-USA100 clone), since this MRSA has replaced the ST239-SCC*mec*III (the Brazilian clone) in this country, and is now the most frequent MRSA lineage in Brazilian hospitals^1^. A high bacterial load (~ 10^9^ CFU/ml) was inoculated on the surface of the TSA plate containing 8 µg/mL vancomycin alone, corresponding to 4MIC, or containing 4MIC vancomycin plus the derivatives **7b** or **7c** at concentrations of 1MIC (128 µg/mL), 1/2MIC (64 µg/mL), 1/4MIC (32 µg/mL), or 1/8MIC (16 µg/mL). The plates were incubated at 37 °C/24 h before being examined. For controlling the activity of the antimicrobials, plates containing vancomycin or each derivative alone at low bacterial load (~10^6^ CFU) were also assayed. Bacterial growth was determined by removing persistent cells from the plates and determining UFC/ml. Controls were also performed using 2% (v/v) DMSO only. Three independent experiments were performed in triplicate.

### Statistical analysis

All experiments were independently performed in triplicate with at least three technical replicates. The results shown are the mean ± standard deviation (SD). Statistical significance was determined by unpaired two-tailed Student’s *t*-test for all experiments, except for the biofilm data that were analyzed by Tukey’s test following One-Way ANOVA, considering a significance level of α = 0.01.

## Results

### Antibacterial activity

The *N*,*O* acetals derived from 2-amino-1,4-naphthoquinone (**6a**), the compounds **7a**, **7b**, and **7c**, showed antimicrobial effects against the representatives of major MRSA international clones (Supplementary Table S2). It is important to highlight the activity of these derivatives—inhibition zones ranging from 8 ± 2.0 to 13 ± 1.0 mm—against the strain BMB9393 (ST239-SCC*mec*III). This MRSA lineage shows resistance to several classes of antimicrobials besides β-lactams, including aminoglycosides, quinolones, and tetracycline^2^, but showed to be  susceptible to all derivatives tested here. The antimicrobial properties of these compounds were also evaluated using the minimal inhibitory concentration (MIC) method. The MIC of the derivatives using broth microdilution for the reference strain ATCC  25923 and MRSA clones ranged between 16 to 128 μg/mL and 64 to 256 μg/mL, respectively (Table 1). The MIC values for the strains BMB9393 (BEC) and CR15-071 (USA100) determined by broth microdilutions were also confirmed by agar dilution method.

**Table 1:** Comparison of the minimum inhibitory concentration (MIC) of the *N*,* O* acetals derived from 2-amino-1,4-naphthoquinone for pandemic MRSA clones, using broth microdilution testing.

Clone (strain)	MIC (µg/mL)			
	**6a**	**7a**	**7b**	**7c**
*Control strain*				
*S. aureus* ATCC 25923	32	128	64	16
*MRSA*				
BEC (BMB9393)	128	256	128	64
NY/Japan-USA100 (CR15-071)	256	256	128	128
USA300 (USA300-0114)	256	128	64	64
USA400 (USA400-0051)	128	128	128	64
OSPC-USA1100 (07-040)	256	256	256	128
Pediatric-USA800 (USA800-0179)	128	256	256	64

### Hemocompatibility profile and cytotoxicity indexes for Vero cells

**Figure 1:**
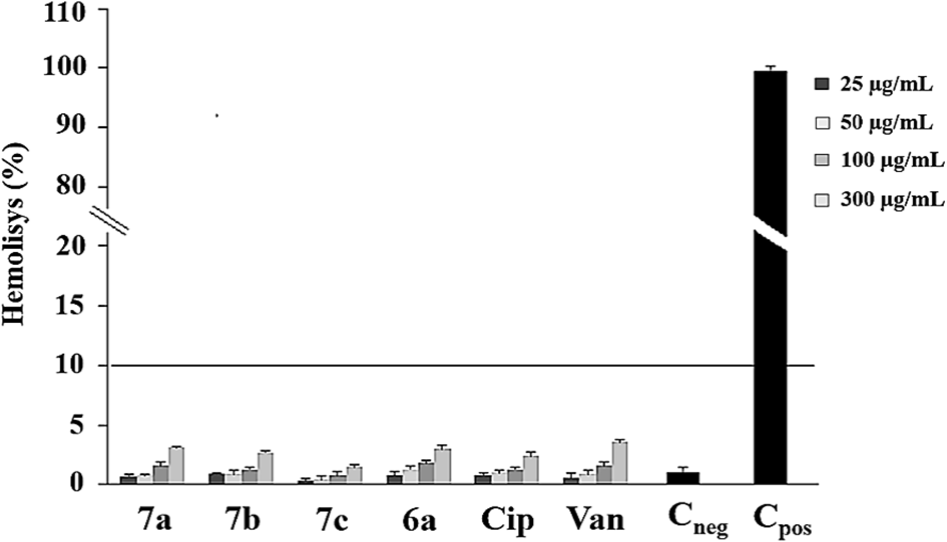
Hemocompatibility profile of **6a** and its derivatives **7a**, **7b**, and **7c** in concentrations of 25 µg/mL, 50 µg/mL, 100 µg/mL and 300 µg/mL. Cip, ciprofloxacin and Van, vancomycin were also used to control this test. C_neg_, negative control (1% DMSO only); C_pos_, positive control (1% Triton X-100).

The hemocompatibility data showed that the derivatives did not interact with erythrocytes membrane showing maximum values of 3.10 ± 0.1% (**7a**), 2.7 ± 0.3% (**7b**), 1.5 ± 0.5% (**7c**), and 3.0 ± 0.9% (**6a**) in the highest concentration evaluated (300 µg/mL) and thus were unable to cause membrane lysis, with no release of hemoglobin (Fig. 1). These values were similar to those of vancomycin (3.6 ± 0.8%) and ciprofloxacin (2.4 ± 0.6%), as shown by t-test.

The cytotoxic concentrations 50% (CC50) for Vero cells showed that **7b** (CC50 = 219.95 ± 1.87) was less toxic compared with **6a**, **7a**, and **7c** derivatives. SI values were calculated to evaluate the selectivity of the derivatives for the bacteria. The **7b** derivative (SI = 1.72) showed some degree of selectivity for eukaryotic cells. Despite that, this value was better than that of sulfamethoxazole-trimethoprim (SI = 1.01) (Table 2).

**Table 2:** Cytotoxicity values (CC_50_), minimum inhibitory concentration (MIC) and selective index (SI) of the *N*,* O* acetals derived from 2-amino-1,4-naphthoquinone.

Derivative	CC50 (µg/mL)	MIC (µg/mL)	SI
**6a**	97.35 ± 3.12	128	0.76
**7a**	155.28 ± 1.03	256	0.61
**7b**	219.95 ± 1.87	128	1.72
**7c**	20.15 ± 2.11	64	0.31
TET	66.6 ± 2.22	32	2.08
TMP-STX	32.58 ± 1.33	32	1.01

*TET* tetracycline, *TMP-STX* trimethoprim-sulfamethoxazole.

### ADMET properties

The *N*,*O*-acetal derivatives were submitted to in silico ADME/Tox (absorption and distribution, metabolism, excretion, and toxicity) analysis (Tables 3 and 4). The theoretical absorption values of **6a**, **7a**, **7b**, and **7c** derivatives were above 70%. However, in the group of the referential drugs, only atovaquone, ciprofloxacin, and furazolidone showed values above 70%, while the data for cefoxitin, doxorubicin, and tetracycline ranged between 30 and 70%, and vancomycin below 30% (Table 3). Most of the derivatives displayed good Caco-2 permeability, similar to atovaquone. Concerning absorption, it was suggested that **6a**, **7a**, **7b**, and **7c** derivatives were not substrates for P-glycoprotein or inhibitors of P-glycoprotein I/II, similarly to furazolidone and nitrofurantoin (Table 3).

**Table 3:** Comparison of ADME (absorption, distribution, metabolism and excretion) parameters between *N*,*O* acetal derivatives from 2-amino-1,4-naphthoquinone and antimalarial (atovaquone) and antibacterial drugs.

Drug	Absorption					Distribution		Metabolism							Excretion	
	Intestinal absorption (human)	Caco-2 permeability	P-glycoprotein substrate	P-glycoprotein I inhibition	P-glycoprotein II inhibition	Vss^a^ (human)	BBB^b^ permeability	Substrate inhibition							Total clearance	Renal OCT2 substrate
								CYP^c^								
								2D6	3A4	1A2	2C19	2C9	2D6	3A4		
	% Absorbed	Categorical (Yes/No)		Categorical (Yes/No)		Numeric (Log L/kg)	Categorical (Yes/No)	Categorical (Yes/No)							Numeric (Log ml/min/kg)	Categorical (Yes/No)
**6a**	74.492	Yes	No	No	No	− 0.086	Yes	No	No	Yes	No	No	No	No	0.299	No
**7a**	80.227	Yes	No	No	No	− 0.156	Yes	No	No	Yes	No	No	No	No	0.424	No
**7b**	95.308	Yes	No	No	No	− 0.094	Yes	No	No	Yes	No	No	No	No	0.446	No
**7c**	94.889	Yes	No	No	No	− 0.039	Yes	No	No	Yes	Yes	No	No	No	0.458	No
Atovaquone	91.413	Yes	Yes	Yes	Yes	0.329	No	No	Yes	Yes	Yes	Yes	No	No	− 0.268	No
Cefoxitin	36.636	No	Yes	No	No	− 1.754	No	No	No	No	No	No	No	No	0.162	No
Ciprofloxacin	96.466	No	Yes	No	No	− 0.17	No	No	No	No	No	No	No	No	0.633	No
Doxorubicin	62.372	No	Yes	No	No	1.647	No	No	No	No	No	No	No	No	0.987	No
Furazolidone	88.575	No	No	No	No	− 0.436	No	No	No	No	No	No	No	No	0.589	No
Nitrofurantoin	79.533	No	No	No	No	− 0.544	No	No	No	No	No	No	No	No	0.655	No
Tetracycline	45.19	No	Yes	No	No	1.194	No	No	No	No	No	No	No	No	0.291	No
Vancomycin	0	No	Yes	No	No	− 0.284	No	No	No	No	No	No	No	No	− 1.273	No

^a^Volume of distribution; ^b^Blood–brain barrier; ^c^Cytochrome P450.

**Table 4:** Comparison of toxicity parameters between *N*,*O* acetal derivatives from 2-amino-1,4-naphthoquinone and antimalarial (atovaquone) and antibacterial drugs.

Drug	Toxicity								
	Oral rat acute toxicity (LD50)	Oral rat chronic toxicity (LOAEL)	hERG I	hERG II	Hepatotoxicity	Toxicological endpoint			
	Numeric (mol/kg)	Numeric (log mg/kg.bw/day)	Categorical (Yes/No)			Immunotoxicity	Carcinogenicity	Cytotoxicity	Mutagenicity
						Categorical (Active/Inactive)			
**6a**	1.776	2.776	No	No	No	Inactive	Active	Inactive	Active
**7a**	2.136	2.512	No	No	No	Active	Active	Inactive	Active
**7b**	2.192	2.503	No	No	No	Inactive	Inactive	Inactive	Active
**7c**	2.158	2.553	No	No	No	Active	Active	Inactive	Inactive
Atovaquone	2.327	2.009	No	Yes	No	Inactive	Inactive	Inactive	Inactive
Cefoxitin	1.544	2.909	No	No	Yes	Inactive	Inactive	Inactive	Inactive
Ciprofloxacin	2.891	1.036	No	No	Yes	Inactive	Inactive	Active	Inactive
Doxorubicin	2.408	3.339	No	Yes	Yes	Active	Inactive	Active	Active
Furazolidone	2.645	1.355	No	No	No	Inactive	Active	Inactive	Active
Nitrofurantoin	2.565	1.390	No	No	No	Inactive	Active	Inactive	Active
Tetracycline	2.214	3.038	No	No	No	Active	Inactive	Inactive	Inactive
Vancomycin	2.482	9.212	No	Yes	Yes	Active	Inactive	Inactive	Inactive

High values for volume of distribution (Vss) − log Vss > 0.45—suggest that the drug will be distributed in body tissue rather than plasma. On the other hand, lower values are considered when log Vss < −0.15. The intermediate values suggest that the drug presents appropriate plasma distribution profile^20^. In this analysis, the compounds showing values below − 0.15 were the derivative **7a**, cefotoxin, ciprofloxacin, furazolidone, nitrofurantoin, and vancomycin. In addition, all derivatives tested were able to pass through blood brain barrier (BBB) according SwissADME webserver (Table 3).

According to the results obtained, none of the compounds evaluated was considered a substrate of CYP2D6, except atovaquone. The derivatives **6a**, **7a** and **7b** did not inhibit CYP2C19, CYP2C9, CYP2D6, and CYP3A4, but showed inhibitory properties against CYP1A2. **7c** derivative did not inhibited CYP2C9, CYP2D6, and CYP3A4, despite their inhibitory effect on CYP1A2 and CYP2C19. Among the referential drugs, only atovaquone showed inhibitory effects on CYP1A2, CYP2C19, and CYP2C9 (Table 3).

The pkCSM webserver was used to analyze the Log (CLtot) values and the possibility of these compounds acting as substrates for organic cation transporter 2 (OCT2). The values of Log (CLtot) of all derivatives ranged from − 1.273 to 0.987, and thus none of them would apparently be a substrate of renal OCT2 (Table 3).

The toxicity parameters of the derivatives studied were predicted using PkCSM and Pro-Tox II webservers (Table 4). The oral rat acute toxicity (LD50) for **6a**, **7a**, **7b** and **7c** derivatives ranged from 1.776 mol/kg to 2.192 mol/kg. The rat oral chronic toxicity (LOAEL) varied from 2.503 log mg/kg b.w. per day to 2.776 mg/kg b.w. per day, and the maximum tolerated dose ranged from 0.516 log mg/kg b.w. per day to 0.778 log mg/kg b.w. per day (Table 4). Hepatotoxicity has been and remains a major reason for drug withdrawal from clinical use^26^. Considering pkCSM prediction, the compounds showing hepatotoxic potential were just amongst referential drugs including cefoxitin, ciprofloxacin, doxorubicin, and vancomycin (Table 4).

Concerning the cardiotoxic potential, all *N*,*O-*acetal derivatives were classified as non-hERG I and II inhibitors. It is noteworthy that atovaquone, doxorubicin, and vancomycin were all classified as hERG II inhibitors, according with platform used (Table 4). Similar to doxorubicin, tetracycline, and vancomycin, the derivatives **7a** and **7c** were predicted to be immunotoxic. Moreover, **6a**, **7a** and **7c** derivatives were expected to be carcinogenic as well as furazolidone and nitrofurantoin. The theoretical mutagenic effect was predicted for the derivatives **6a**, **7a** and **7b**. However, this effect was also observed for doxorubicin, furazolidone, and nitrofurantoin, which are drugs of current clinical use (Table 4).

### Impact of *N*,*O* acetals derived from 2-amino-14-naphthoquinone on biofilm formation

**Figure 2:**
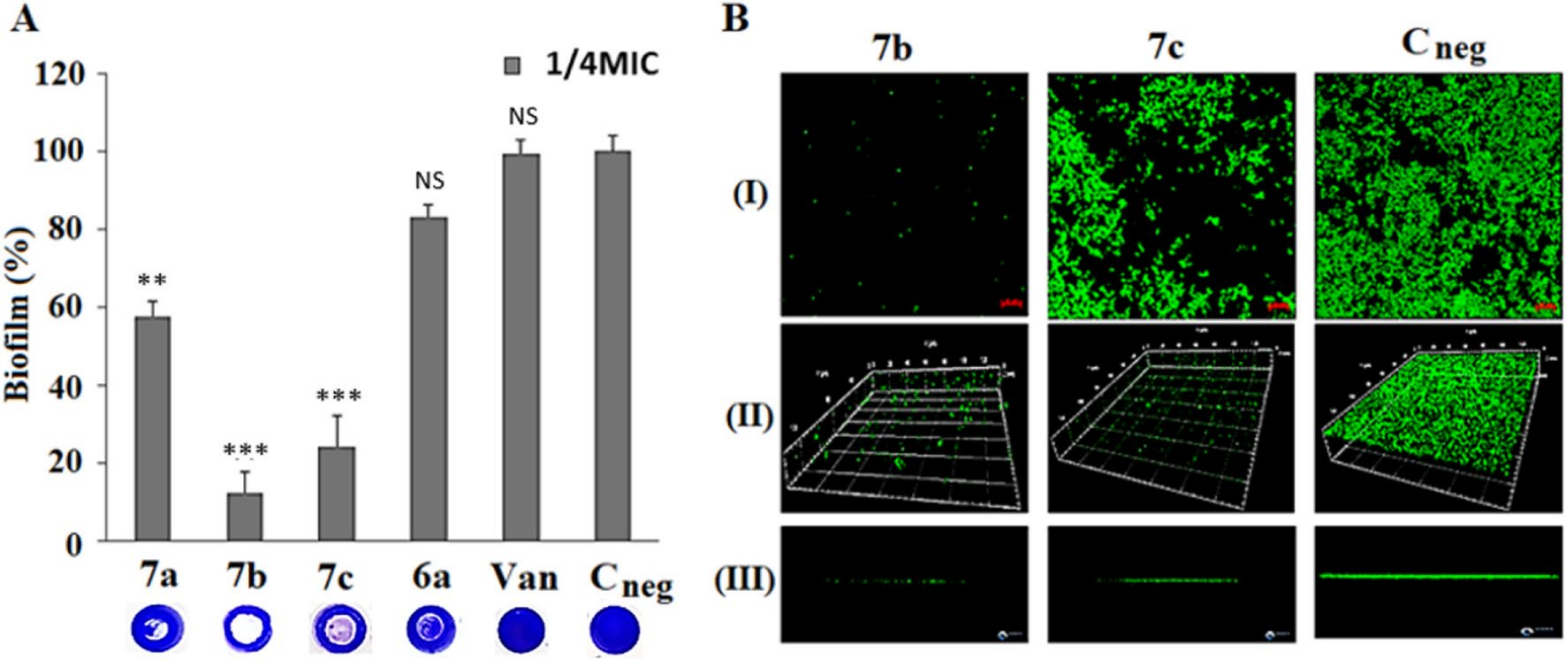
Effect of 1/4MIC of **6a** and *N*,*O* acetal naphthoquinone derivatives **7a**, **7b**, and **7c** on biofilm development. (**A**) Biofilm formed on surfaces and stained with crystal violet. (**B**) Confocal laser scanning microscopy (CLSM). Effects of 1/4MIC of the derivatives **7b** (32 µg/mL) and **7c** (16 µg/mL) on biofilm formed by the MRSA strain BMB9393, compared with the untreated biofilm (C_neg_). *Van* vancomycin (1/4 MIC; 0.5 µg/mL). *NS* not significant; **p value < 0.001; and ***p < 0.0001. The biofilms were treated with SYTO 9 and the image obtained in (**I**) two-dimensional axis (xy) and (**II**) three-dimensional plane (xyz). (**III**) Biofilm thickness. Red scale = 10 μm. For each set of experiments, a total of three independent experiments were performed in quadruplicate.

The concentration of 32 μg/ml (1/4MIC) of **7b** was enough to reduce the accumulated biofilm matrix by 87.4% ± 1.9% while 16 μg/mL (1/4MIC) of **7c** reduced by 75.6% ± 3.7%. This inhibition was more specific for biofilm development since such a strong impact in growth reduction was not observed for planktonic cells at these concentrations (Supplementary Figure S2). In addition, 1/4MIC **6a** (32 μg/ml) and **7a** (64 μg/ml), although showing antimicrobial effects, were much less effective at reducing biofilm development compared with **7b** or **7c** (Fig. 2A). It is notable that vancomycin, one of the last resources to treat MRSA severe infections, was not able to reduce biofilm development (Fig. 2A). CLSM was used to visualize the biofilm architectures and clearly document the inhibitory effects of 1/4MIC **7b** and **7c** (Fig. 2B). The images obtained endorse the results observed in a microplate-based assay, demonstrating that **7b** and **7c** were able to decrease biofilm accumulation massively compared with untreated biofilms (Fig. 2B). In these assays, **7b** also showed superior ability in reducing biofilm formation in relation to **7c** (Fig. 2B).

### The antibiofilm effect of *N*,*O*-acetals derived from 2-amino-14-naphthoquinones was linked to decreased expression of biofilm-associated genes

**Figure 3:**
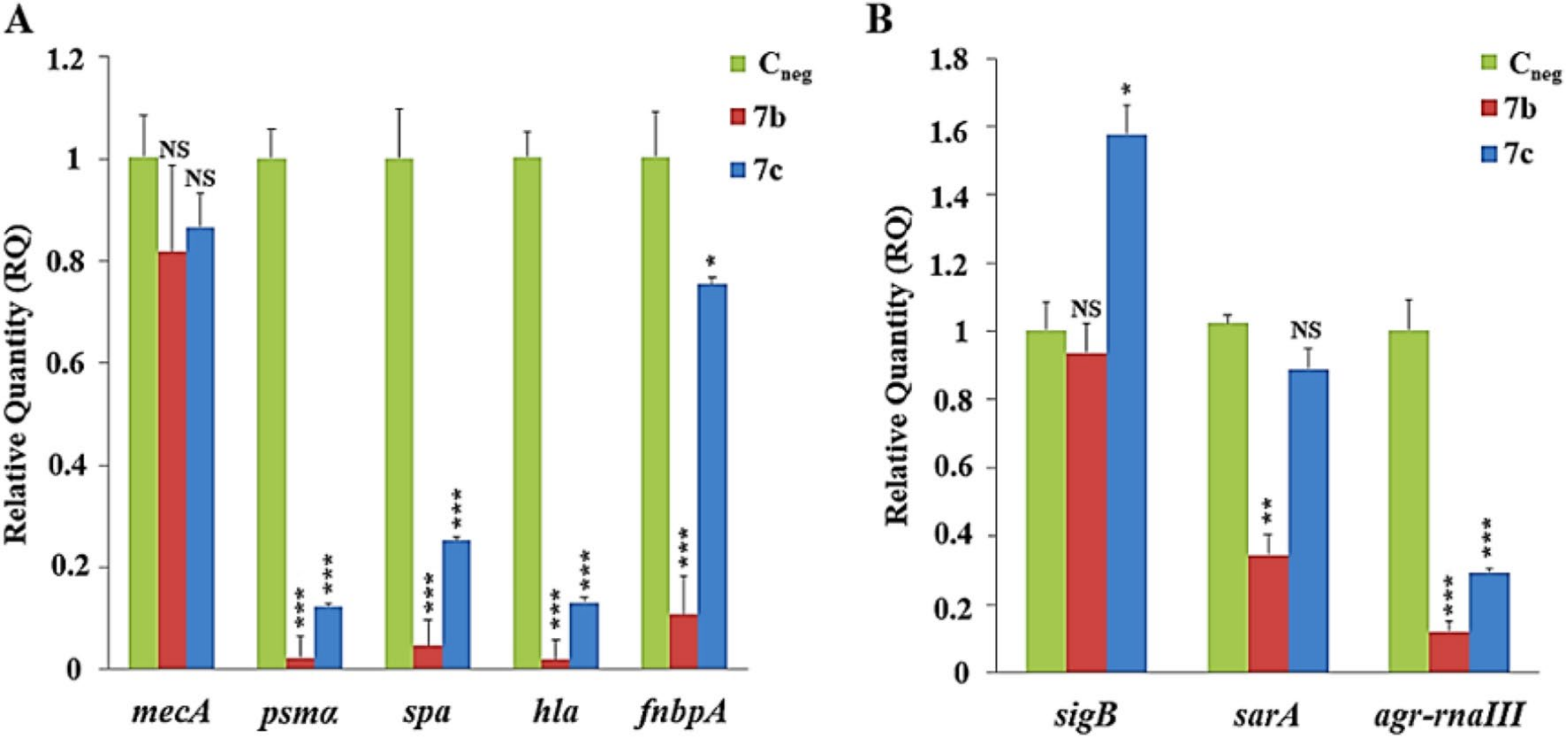
Effect of **7b** and **7c** derivatives in the expression of the biofilm-associated genes and global gene regulators. Gene expression was assessed by real-time qRT-PCR after treatment of the MRSA strain BMB9393 with 1/4MIC of derivatives **7b** (32 µg/mL) or **7c** (16 µg/mL). (**A**) Gene expression for biofilm-associated genes *fnbA*,* hla*,* spa*,* psmα3*, and *mecA*. (**B**) Gene expression for the biofilm regulators *rnaIII*, *sarA*, and *sigB*. Data were represented by the mean of three independent experiments with triplicates. The bar represents the standard deviation. C_neg_, negative control (2% DMSO, only). *NS* not significant; *p values < 0.01; **p < 0.001; and ***p < 0.0001.

Some *S. aureus* genes are well known for their role in biofilm development. Therefore, due to the strong biofilm inhibition promoted by the derivative **7b**, we investigated the role played by this compound in the expression of important biofilm-associated genes, to get some insights on the mechanisms by which this molecule strongly impairs biofilm production. Additionally, the derivative **7c**, which also affects biofilm accumulation, but in lesser extent, was also included for comparison purpose. Among these genes, the *mecA* encoding PBP2A was not significantly affected by 1/4MIC of **7b** (32 µg/mL) or **7c** (16 µg/mL) (Fig. 3A). The expression of *fnbA* encoding FnbpA protein—an important adhesin in *S. aureus* biofilm development—was reduced by 89.3% ± 1.5% after treatment with derivative **7b** (*p* < 0.0001). However, for the **7c** derivative, the reduction for this gene was only 24.64% ± 3.5% (*p* < 0.01) (Fig. 3A). The expression of *hla* encoding hemolysin/α-toxin (Hla) was considerably reduced by 90.0 ± 0.6% and 86.5 ± 1.0% after treatment with derivatives **7b** and **7c**, respectively (*p* < 0.0001). Also, an important reduction—by 95.4 ± 0.9% for **7b** and 74.82 ± 4.9% for **7c** (*p* < 0.0001)—was observed for the *spa* gene, which encodes the staphylococcal protein A. The expression of another important biofilm-associated gene, the *psmα3* gene encoding the phenol soluble modulin alpha 3—was reduced by 97.75 ± 0.6% for **7b** and 87.73 ± 4.3% for **7c** (*p* < 0.0001) (Fig. 3A).

### The role of global staphylococcal regulators in the decreased expression of biofilm-associated genes

The effect of 1/4MIC of the derivatives **7b** (32 µg/mL) and **7c** (16 µg/mL) in the expression of *rnaIII* (encoding the RNAIII, the main effector molecule of the quorum-sensing Agr), *sarA* (encoding the transcriptional regulator SarA), and *sigB* (encoding an alternative RNA polymerase sigma subunit) were tested (Fig. 3B). A pronounced decrease was observed for the *rnaIII* expression, which regulates the temporal expression of several *S. aureus* virulence genes, such as *hla*, *spa*, *fnbpA* and *psmα3*. The number of RNAIII transcripts was reduced by 88.0 ± 1.1% for derivative **7b** and 70.6 ± 2.2% for **7c** (*p* < 0.0001). Derivative **7b** also reduced importantly *sarA* gene expression at 65.5 ± 6.0% (*p* < 0.001). However, no important effect on *sarA* expression was observed for the derivative **7c**. Moreover, derivative **7c** increased *sigB* transcripts at 57.22 ± 5.2% *(p* < 0.01) while **7b** did not affect the expression of the *sigB* transcription factor (Fig. 3B).

### Effect of *N*,*O*-acetals derived from 2-amino-1,4-naphthoquinone in the *S. aureus* autolytic system

**Figure 4:**
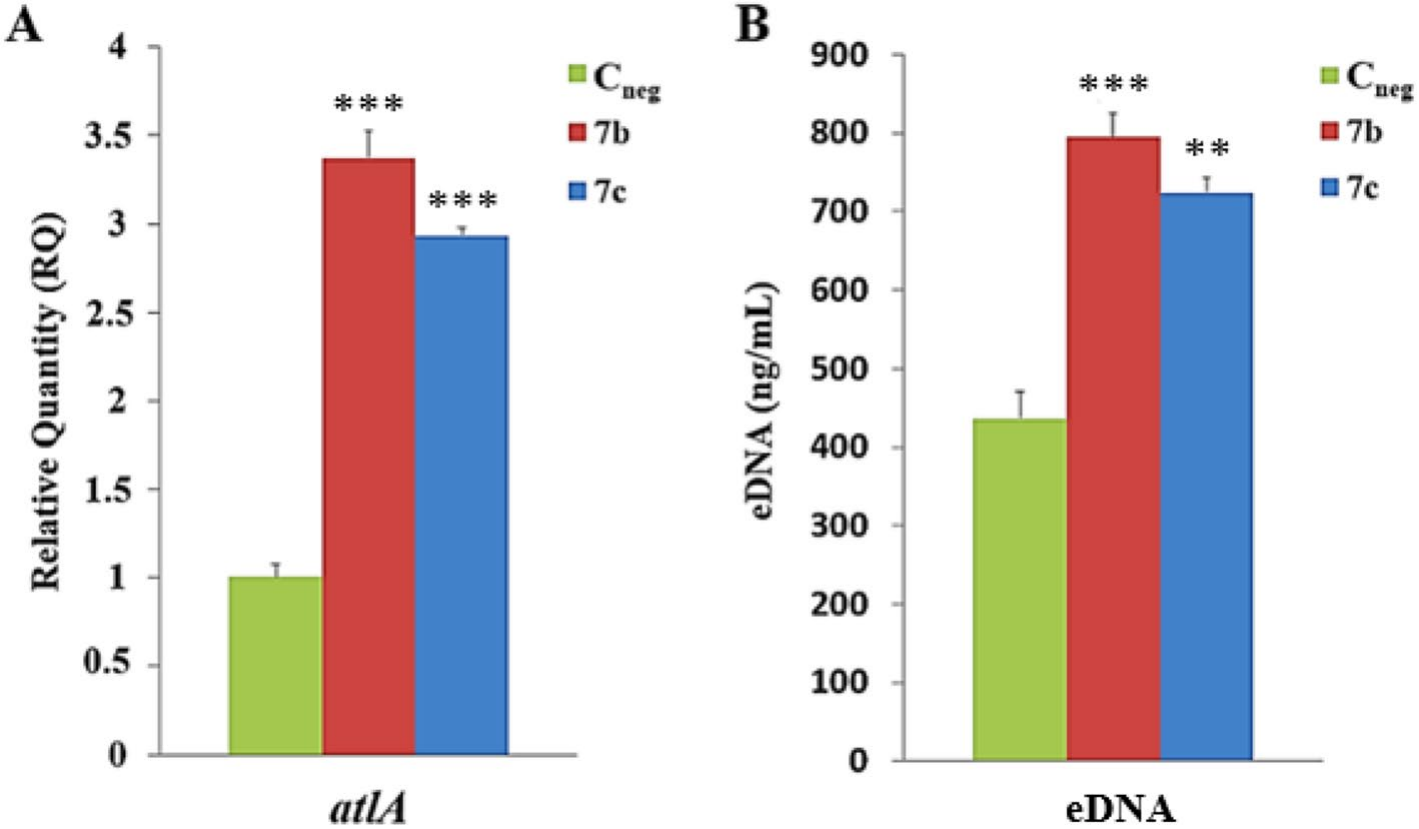
Effect of derivatives **7b** and **7c** on the expression of the major *S. aureus* autolysin (AtlA) using the MRSA strain BMB9393. (**A**) *atlA* expression was evaluated by real-time qRT-PCR after-treatment of the MRSA strain BMB9393 with 1/4MIC of derivatives **7b** (32 µg/mL) and **7c** (16 µg/mL). (**B**) eDNA was determined after-treatment of the MRSA strain BMB9393 with 1/4MIC of derivatives **7b** and **7c**. Data represent the mean of three independent experiments with triplicates. The bar represents the standard deviation. C_neg_, negative control (2% DMSO only). *NS* not significant; **p values < 0.001 and ***p < 0.0001.

The role played by 1/4MIC **7b** (32 µg/mL) on the autolytic activity was evaluated in the biofilm cell growth of the strain BMB9393. An important increase of 3.5-times (*p* < 0.0001) in the *atlA* transcripts was observed. This result was validated by the fact that 1/4MIC **7c** (16 µg/mL) led to three-fold increase in the *atlA* expression (*p* < 0.0001) (Fig. 4A). In agreement with this data, the amount of eDNA measured in the bacterial supernatant increased, after treatment of bacterial cultures with **7c**. Indeed, the bacterial cell lysis was even higher (about two-fold) for the derivative **7b** (*p* < 0.0001), which triggered an increased expression of *atlA* in comparison with **7c** (Fig. 4B).

### Evaluation of *N*,*O*-acetal derivatives against MRSA cells persistent to vancomycin

**Figure 5:**
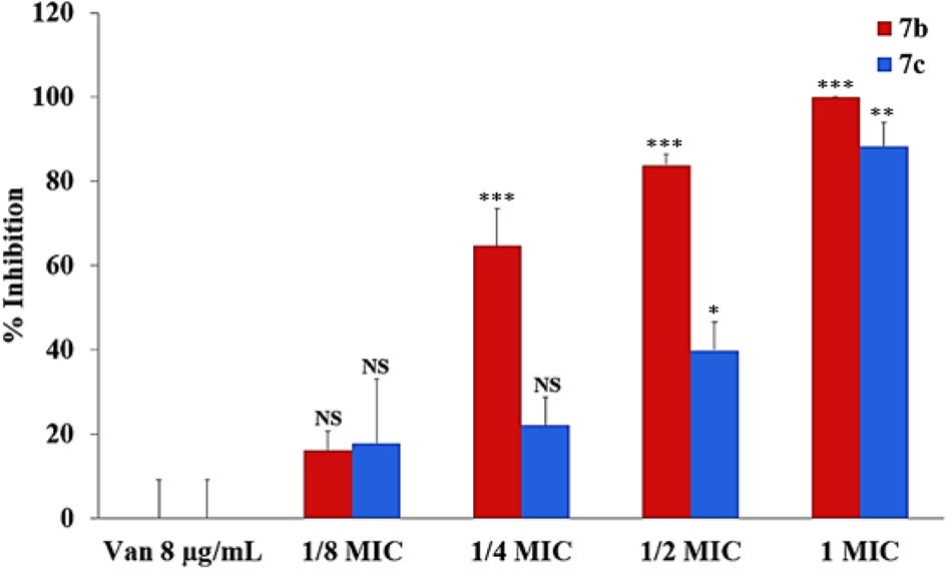
Effect of *N*,*O*-acetal derivatives against MRSA cells persistent to vancomycin. Persistent cells of the strain related to USA100 clone (CR15-071) were detected in plates containing 8 µg/mL vancomycin and high bacterial load. A dose dependent inhibition of persistent cells was observed for different combinations of vancomycin (8 µg/mL) with concentrations of 1/8MIC (16 µg/mL) to 1/2MIC (64 µg/mL) of each derivative. Note that 1MIC (128 µg/mL) of the derivative **7c** was able to eliminate vancomycin-persistent cells completely. Data were represented by the mean of three independent experiments with triplicates. The bar represents the standard deviation. *NS* not significant; *p < 0.01; **p < 0.001; and ***p < 0.0001.

It is well known that harsh environments such as those encountered in biofilm growth, nutrient-depleted conditions and other stressful conditions can lead to the emergence of nonresistant bacteria that are refractory to antimicrobial treatment. In this study, high inoculum size was used to generate subpopulations of vancomycin-persistent cells at concentrations as high as 4MIC (8 µg/mL). After 18 h incubation, refractory/persistent cells (4.5 × 10^8^ CFU/mL) of the strain (CR15-071) representative of the clone USA100 were recovered from vancomycin plates (4MIC). However, when 1MIC of the derivatives **7b** (128 µg/mL) or **7c** (128 µg/mL)—both effectives to inhibit biofilm development—were added to the plates containing vancomycin (4MIC), a complete inhibition of persistent cells was achieved for derivative **7b** (*p* < 0.0001), and an important reduction of 88.3% (*p* = 0.0002) in the total of persistent cells were caused by the association with the derivative **7c** (Fig. 5). Accordingly, the *N*,*O* acetal derivative **7b** was not only able to inhibit bacterial biofilm, but also showed an important and impressive ability to kill persistent cells at MIC concentrations, which were totally refractory to 4MIC vancomycin.

## Discussion

The *N*,*O* acetals derived from 2-amino-1,4-naphthoquinone, **7a**, **7b**, and **7c** showed antibacterial activity not only for the methicillin-susceptible strain but also for internationally spread MRSA lineages, including ST239-SCC*mec*III, which shows high multiresistance level, homogeneous resistance to methicillin, and increased ability to accumulate biofilm^2^. Divergent MIC values were observed for some of these derivatives in relation to previous studies with the strain ATCC 25923^11^. However, this may be attributed to the use of different methods in these two studies. Although we did not compare broth microdilution with broth macrodilution method, the latter used in the previous study^11^, the MICs for the derivatives **7a**, **7b**, **7c** performed here, using Mueller Hinton agar dilution, confirmed the MIC data obtained by broth microdilution of the MRSA strains BMB9393 (BEC) and CR15-071 (USA100). Actually, previous studies have already demonstrated disagreement in MIC data obtained with different recommended methods for some drug/strain combinations, including for *S. aureus*^27^. The MRSA lineages (ST1-SCC*mec*IV, ST5-SCC*mec*II, ST8-SCC*mec*IV, ST30-SCC*mec*IV, and ST239-SCC*mec*III) tested for these derivatives are currently causing the majority of hospital and community infections worldwide, including bacteremia and biofilm-associated infections^1^. Notably, despite the infection control measures, bacteremia caused by MRSA remains at high frequency and accounts for a mortality rate of about 25%^28^.

The in vitro toxicity assays revealed that the derivatives **7a**, **7b**, and **7c** did not display a hemolytic profile, according to Dobrovolskaia and collaborators^19^. The toxicity tests using monolayers of the Vero cell line revealed that the **7b** derivative showed better SI value indicating that **7b** displayed not only antibacterial effect but also higher selectivity against MRSA strain than toxicity to eukaryotic cells. Theoretical pharmacokinetic properties are important parameters to assess potential compounds during drug discovery process. All *N*,*O-acetal* derivatives showed good predictions for absorption values in the human intestine, similar to atovaquone, ciprofloxacin, and furazolidone. Indeed, the compounds **7b** and **7c** showed absorption values greater than 90%, close to that of ciprofloxacin, which is a good estimative for oral bioavailability^29^.

Interestingly, only *N*,*O*-acetal derivatives and atovaquone showed permeability to Caco-2 cells, which has been used as a model for human intestinal absorption of drugs^30^. The results also suggested that none of the *N*,*O*-acetal derivatives seem to affect P-glycoprotein, which is involved in drug exclusion^31^. All together, these data reveal an optimal in silico prediction of oral bioavailability for the *N*,*O*-acetal derivatives. Derivatives **6a**, **7b** and **7c** showed intermediate Vss values, representing good predictions for adequate plasma distribution profiles. In addition, the analysis of BBB values, according to SwissADME calculation, suggested that *N*,*O-acetal* derivatives could cross blood brain barrier^32^.

It has been estimated that CYPs could be able to metabolize around 75% of the commercially available drugs^33^. None of the derivatives tested was substrate for cytochrome P450 isozyme CYP2D6 and CYP3A4. Moreover, these derivatives were only presumed to be inhibitors of CYP1A2, except **7c** derivative that was predicted to inhibit CYP2C19. Interestingly, CYP1A2 is one main xenobiotic metabolizing enzyme in humans, and a recent study associated this enzyme with the bioactivation of procarcinogens, including 4-(methylnitrosamino)-1-(3-pyridyl)-1-butanone (NNK), a tobacco specific and potent pulmonary carcinogen^34^.

Drug clearance was measured by determining Log(CLtot). Except for **6a**, all derivatives tested presented high Log(CLtot) values, but still acceptable. Actually, these values were lower than those observed for referential drugs such as ciprofloxacin, doxorubicin, furazolidone, and nitrofurantoin. None of the *N*,*O-acetal* derivatives tested was predicted to show hepatotoxicity. Also, they were not expected to inhibit hERG I and II channels. Drug-hERG channel interactions have been considered a therapeutic challenge as a major cause acquired-long QT syndrome. Actually, a relationship between chronic heart failure (CHF) and *S. aureus* bacteremia was suggested. CHF patients with *S. aureus* bacteremia showed a significantly higher mortality rate compared with patients with normal heart functions^35,36^. This observation reinforces the need of non-cardiogenic effect for anti-MRSA drug candidates and highlights this promising profile of our derivatives.

Data from the ProTox-II platform revealed that the **7b** derivative showed good toxicological parameters, except for the mutagenic profile. This feature could be related to the mechanism of action of these derivatives. Besides oxidative stress, another mechanism of action generally associated with naphthoquinones is the ability to bind to topoisomerase complex causing damage to the DNA replication process^37^. Despite that, in vitro studies using V79 Chinese hamster cells did not confirm a mutagenicity effect for 1,4-naphthoquinone^38^.

Besides the antimicrobial activity against MRSA and the improvement of pharmacological parameters, the introduction of the 2-ethoxymethyl radical generating the **7b** derivative resulted in the best antibiofilm and antipersistence effects. Furthermore, contrarily to the substitution by 2-methoxymethyl or 2-propynyloxy, the 2-ethoxymethyl substituted amino-1,4-naphtoquinone did not inhibit four of five CYPs enzymes tested, was not a substrate of CYP3A4 and CYP2D6, and also was predicted to have the best intestinal absorption, comparable to ciprofloxacin.

It is well known that biofilm development enhances *S. aureus* ability to cause infections and persist into the host^5^. In the present study, the *N*,*O* acetal derivatives from 2-amino-1,4-naphthoquinone, **7b** and **7c**, were able to reduce biofilm accumulation in 88% and 75%, respectively, at 1/4MIC without causing similar massive reduction in viability of planktonic cells. Oja and collaborators^39^ also found that other naphthoquinone derivatives [biosynthetic pyranonaphthoquinone (PNP) polyketides] impaired biofilms formed by methicillin-susceptible *S. aureus* (MSSA), ATCC 25923, and Newman strains. It is important to remark that vancomycin, considered one of the last resources to treat serious infections by MRSA, did not impair biofilm development in the study model chosen. Not only that, induction of biofilm by vancomycin had already been observed for some *S. aureus* strains^40^.

In one of the first stages of biofilm development, bacteria adhere to biotic or abiotic surfaces, in a mechanism mediated by bacterial surface adhesion molecules, such as the fibronectin-binding protein A (FnBPA) encoded by the *fnbA* gene^5^. The expression of *fnbA* is upregulated by the *sarA* in an *agrRNAIII*-independent mode^41^. The derivative **7b**, that showed a drastic reduction in biofilm accumulation in 1/4MIC, also presented a deeper decrease in the expression of the *fnbA* gene paralleled by an also important decline in *sarA* transcripts. Actually, it is well known that inhibition of *sarA* also leads to impairment of biofilm development in *S. aureus*^42^, which might explain the important reduction observed in the biofilm accumulation caused by the **7b** derivative. In agreement with these data, the decrease of *fnbA* transcripts for MRSA treated with **7c** was less pronounced when compared with **7b**. Indeed, **7c** also presented a lower ability to impair biofilm development compared with **7b**. Moreover, the poorer effect of **7c** in the decreasing *sarA* expression could be explained by the increase in the expression of *sigB* caused by **7c**. SigB is a *sarA*-positive regulator that has also been implicated in the modulation of the maturation phase of *S. aureus* biofilms^43,44^.

Protein A is an antiphagocytic protein encoded by the *spa* gene that can also provide *S. aureus* with self-aggregation property, which is an important condition for biofilm development^45,46^. The MRSA treatment with both **7b** and **7c** derivatives caused an important reduction in the *spa* expression, which was more pronounced for **7b**, and thus it might also account for the stronger biofilm impairment caused by these derivatives. The control of *spa* expression is complex and involves an intricate regulatory network^47^.

Notably, the derivatives **7b** and **7c** showed antibiofilm effects associated not only with a strong inhibition of *fnbA* and *spa* genes but also with an important attenuation in the expression of cytotoxicity-associated genes, such as *hla* encoding the staphylococcal α-hemolysin, *hld* encoding δ-hemolysin (whose coding sequence is localized inside the sequence of *agr*-*rnaIII*), and *psmα3* encoding phenol-soluble modulin alpha 3 peptide^47,48^. It is very well known that PSMs are multifactorial molecules in staphylococcal pathogenesis. PSMs are broadly cytolytic, inducing the killing of different cell types. Furthermore, PSMs are important for the architecture of the *S. aureus* biofilms^49^. The α-hemolysin (Hla) is the prototype for small β-barrel pore-forming cytotoxins, which induces lysis of different host cells, including monocytes, neutrophils, pneumocytes, and endothelial cells. Besides its action promoting biofilm development^50^, it was observed that Δ*hla* mutants lead to attenuated bloodstream infections in animal models^51^. It is notable that the inhibitory effects in toxin-associated genes were also more pronounced for **7b** than **7c**. Due to the importance of these genes for *S. aureus* pathogenesis, although we did not perform studies using animal models, it seems logical to suppose that the attenuation of cytotoxic genes, caused by these derivatives, is likely to result in antivirulence effects^49,51^.

It was previously found that *agr*-RNAIII can positively or negatively regulate biofilms depending on the background of the strains, and that *agr*-RNAIII is a positive biofilm regulator for strains with the ST239-SCC*mec*III background, such as BMB9393^24^. In fact, *agr*-RNAIII was impaired by both derivatives **7b** and **7c**, and again **7b** caused a deeper effect by diminishing RNAIII transcripts. The impairment of RNAIII agrees with the reduction observed in *hla* and *psmα3*, since RNAIII is a positive regulator for these genes. AtlA is the main *S. aureus* muramidase that has an important role in cell wall biogenesis and the septation process during bacterial cell division^52^. It was found that small fractions of bacterial cell lysis enhance biofilm formation due to the ligation of eDNA to proteins and polysaccharides of the biofilm matrix^53^. However, it was demonstrated that the uncontrolled expression of *atlA* causes increased cell lysis, resulting in a defective biofilm formation^54^. Thus, the triggering of the autolytic system by **7b** and **7c** is likely to play a role in the impact of these naphthoquinone derivatives in MRSA biofilm development.

Therapeutic failures during the administration of vancomycin have been described, and in some cases, involved patients with MRSA infections^55^. A hypothesis suggested to explain these failures was linked to a decreased penetration of vancomycin into the biofilm matrix^56,57^. Also, it was proposed that stress conditions in the hyper-populated environment, such as those encountered in biofilm growth or high bacterial load, might induce the generation of persistent cells^58^. However, the exact mechanisms through which bacteria become refractory/persistent to different antimicrobials in the biofilm environment are still to be completely clarified^5^.

In this study, we found that **7b** derivative at 1MIC (128 µg/mL) was able to eradicate subpopulations of persistent cells that survived to concentrations of 4MIC (8 µg/mL) vancomycin. The ability of these derivatives to trigger cell autolysis might be involved in the killing of persistent cells. It was previously shown that the deletion of the operon *msaABCR* of *S. aureus* increased the processing of the major *S*. *aureus* autolysin by proteases, leading to the activation of the muramidase activity. In addition, it was found that *msaABCR* deletion enhanced the effectiveness of antibiotics against persistent cells, which was attributed to the increased cell lysis^54^. Thus, it is interesting to further investigate the role played by the autolytic system in the killing of vancomycin-persistent cells by these naphthoquinone derivatives and other active molecules.

## Conclusions

**Figure 6:**
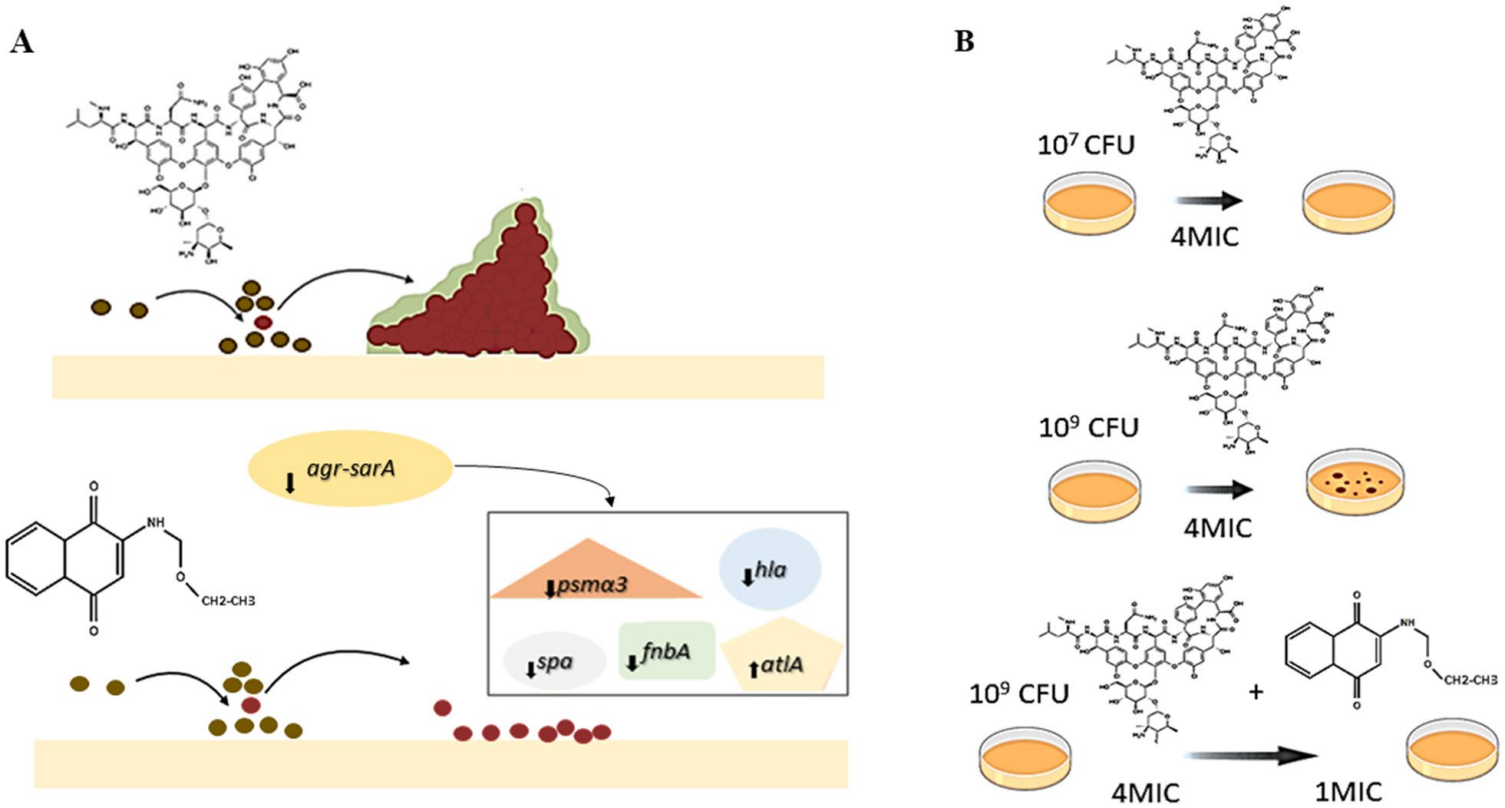
Antibiofilm and antipersistence effects of the *N*,*O* acetal derivative **7b** in MRSA. (**A**) Upper panel: In concentrations as high as 4MIC (8 µg/mL), vancomycin does not impair biofilm formation. (**A**) Bottom panel. Contrarily to vancomycin, 1/4MIC derivative **7b** (32 µg/mL) reduced biofilm development, importantly. The inhibition of biofilm formation was followed by reduced expression of the global virulence regulators *agr* and *sarA*, with concomitant inhibitions of biofilm-associated genes including *fnbA* and *psmα3*, among others. (**B**) Vancomycin-persistent cells formed at condition of high bacterial load (10^9^ CFU) could be eliminated by the association of derivative **7b** to vancomycin in the culture media.

In conclusion, the accelerated evolution and dissemination of antimicrobial resistance in *S. aureus* are outpacing the development of completely new antibiotics, and antibiofilm agents might provide an interesting alternative. In this context, the 2-(ethoxymethyl)-amino-1,4-naphthoquinone (**7b**) derivative, besides the antimicrobial action, also showed strong antibiofilm and antipersistence effects against multidrug-resistant MRSA strains. These effects were paralleled by the ability of **7b** to concomitantly affects (directly or indirectly) important *S. aureus* virulence regulators (*agr*-RNAIII and *sarA*) and biofilm-associated genes (*spa*, *fnbA*, *hla* and *psmα3*). Finally, these properties associated with the biological and pharmacological aspects of this compound identified **7b** derivative as an interesting model for the design of potentially promising and more effective drugs against MRSA (Fig. 6).

## Supplementary information

Supplementary information.
