# Supplementary material for: Antibiofilm effects of N,O-acetals derived from 2-amino-1,4-naphthoquinone are associated with downregulation of important global virulence regulators in methicillin-resistant Staphylococcus aureus
Source: Sci Rep. 2020 Nov 12;10:19631. doi: 10.1038/s41598-020-76372-z (PMC7661526; doi:10.1038/s41598-020-76372-z)
Supplement: Supplementary file 1 — Supplementary information. [file 41598_2020_76372_MOESM1_ESM.docx]

Antibiofilm effects of *N,O*-acetals derived from 2-amino-1,4-naphthoquinone are associated with downregulation of important global virulence regulators in methicillin-resistant *Staphylococcus* *aureus*

Juliana Silva Novais^1^, Mariana Fernandes Carvalho^1^, Mariana Severo Ramundo^2^, Cristiana Ossaille Beltrame^2^, Reinaldo Barros Geraldo^1^, Alessandro Kappel Jordão^3^, Vítor Francisco Ferreira^4^, Helena Carla Castro^1*^ & Agnes Marie Sá Figueiredo^1,2*^

^1^Universidade Federal Fluminense, Programa de Pós-graduação em Ciências e Biotecnologia, Instituto de Biologia, Niterói, 24210130, Brazil

^2^Universidade Federal do Rio de Janeiro, Instituto de Microbiologia Professor Paulo de Góes, Departamento de Microbiologia Médica, Rio de Janeiro, 21941902, Brazil

^3^Universidade Federal do Rio Grande do Norte, Centro de Ciências da Saúde, Departamento de Farmácia, Natal, 59012-570, Brazil

^4^Universidade Federal Fluminense, Departamento de Química Orgânica, Instituto de Química, Campus do Valonguinho, Niterói, 24020-007, Brazil

*Corresponding author. H. C. Castro: Instituto de Biologia, Universidade Federal Fluminense, Campus Valonguinho, 24210-130. Niterói, RJ, Brazil. Tel:+55 21 26299954. E-mail hcastrorangel@yahoo.com.br. A. M. S. Figueiredo: Instituto de Microbiologia Paulo de Góes, Universidade Federal do Rio de Janeiro, Departamento de Microbiologia Médica, Bloco I, Cidade Universitária, 24220-590, Rio de Janeiro, RJ, Brazil. Tel: +55 21 22604193. E-mail: agnes@micro.ufrj.br.


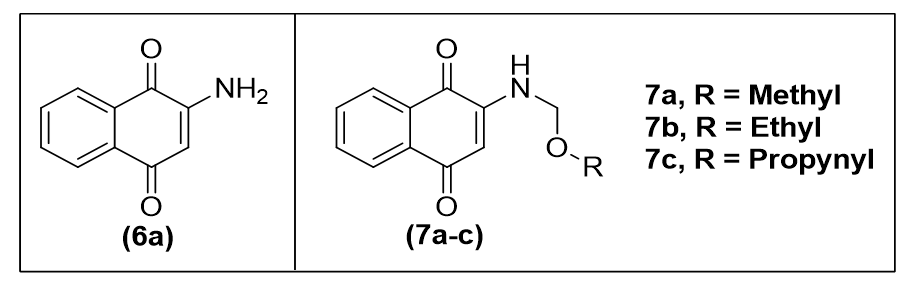


**Supplementary Figure S1**. Chemical structure of the *N,O* acetals **7a**, **7b**, and **7c** derived from 2-amino-1,4-naphthoquinone (**6a)**.


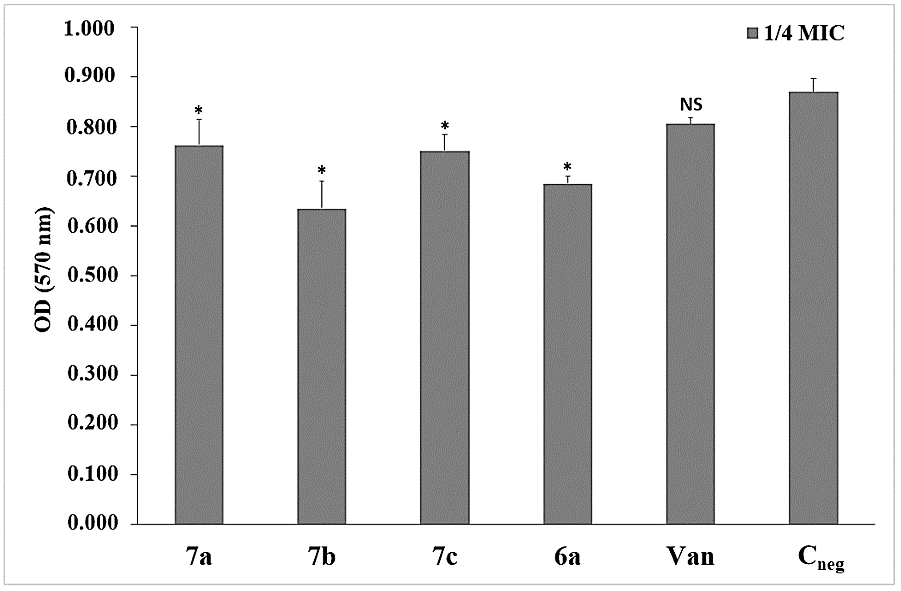


**

***

***

**

**Supplementary Figure S2**. Effect of 1/4MIC *N,O*-acetals **7a** (64 µg/mL)**, 7b (**32 µg/mL)**, 7c (**16 µg/mL) derived from 2-amino-1,4-naphthoquinones **6a** (32 µg/mL), and 1/4MIC vancomycin ( 0.5 µg/mL) on cell growth of the strain MRSA strain BMB9393. **Cneg**, negative control with 2% DMSO only. NS: not significant; **: *p* value < 0.001; ***p<0.0001. Colony forming units (CFU) were also determined to confirm the viability of the cells grown in the presence of 1/4MIC of the derivatives **7b (**1.60 X 10^8^ CFU/mL) and **7c (**3.53 X 10^8^ CFU/mL), which displayed important antibiofilm effects, and also for the negative control: **Cneg** (4.53 x 10^8^ CFU/mL).

**Supplementary Table S1.** Primers used in this study.

| **Gene** | **Gene product** | **Primer** | **Reference** |
| --- | --- | --- | --- |
| *gyrA* | DNA gyrase subunit A | F: GCAGTTCGCTTAACAAGTGG | This study |
|  |  | R: GCCGTACAGCAACGGGTG |  |
| *gyrB* | DNA gyrase subunit B | F: GATGTAGCAAGTCTTCCAGG | This study |
|  |  | R: ACAAAATCTGGTCGTGACTC |  |
| *atlA* | Autolysin | F: TGTCGAAGTATTTGCCGACTTCGC | 1 |
|  |  | R: TGGAATCCTGCACATCCAGGAAC |  |
| *fnbA* | Fibronectin binding protein A | F: ACTTGATTTTGTGTAGCCTTTTT | 2 |
|  |  | R: GAAGAAGCACCAAAAGCAGTA |  |
| *hla* | Alpha-toxin | F: TTTGTCATTTCTTCTTTTTCCCA | 2 |
|  |  | R: AAGCATCCAAACAACAAACAAAT |  |
| *spa* | Protein A | F: TGGTTTGCTGGTTGCTTCTTA | 2 |
|  |  | R: GCAAAAGCAAACGGCACTAC |  |
| *pmsα*3 | Phenol soluble modulin *α*3 | F: TATCAAAAGCTTAATCGAACAATTC | 3 |
|  |  | R: CCCCTTCAAATAAGATGTTCATATC |  |
| *mecA* | Penicillin binding protein 2a | F: TCCAGATTACAACTTCACCAGG | 4 |
|  |  | R: CCACTTCATATCTTGTAACG |  |
| *rnaIII* | Agr *rnaIII* | F: AATTTGTTCACTGTGTCGATAAT | 2 |
|  |  | R: TGGAAAATAGTTGATGAGTTGTT |  |
| *sarA* | Transcriptional regulator SarA | F: TTCTTTCTCTTTGTTTTCGCTG | 2 |
|  |  | R: GTTATCAATGGTCACTTATGCT |  |
| *sigB* | Sigma factor | F: TCAGCGGTTAGTTCATCGCTCACT | 5 |
|  |  | R: GTCCTTTGAACGGAAGTTTGAAGCC |  |
| 16S *rrna* | Reference gene | F: AGAGATAGAGCCTTCCCCTT | 2 |
|  |  | R: TTAACCCAACATCTCACGACA |  |

| **Supplementary Table S2.** Disk diffusion test for *N,O* acetals derived from 2-amino-1,4-naphthoquinone using reference strain and MRSA strains from different international lineages. | | | | | | |
| --- | --- | --- | --- | --- | --- | --- |
| **Strain** | | **Inhibition zone (mm)** | | | | |
|  |  | **6a** | **7a** | **7b** | **7c** | **VAN*** |
| Control strain | *S. aureus* ATCC 25923 | 9±1.4 | 9.7±2.0 | 12.5±3.5 | 14±3.4 | 13±1.0 |
| MRSA | ST239-SCC*mec*III  (BMB9393) | 8±2.0 | 10±1.5 | 10±1.0 | 13±1.0 | 14±1.0 |
|  | ST5-SCC*mec*II  (USA100) | 9±1.0 | 12±1.0 | 13±2.0 | 12±1.5 | 16±1.5 |
|  | ST8-SCC*mec*IV  (USA300) | 9±1.0 | 14±1.0 | 12±2.0 | 13±1.3 | 17±1.0 |
|  | (ST1-SCC*mec*IV)  (USA400) | 8±1.2 | 13±2.0 | 13±1.0 | 11±0.8 | 16±2.0 |
|  | ST30-SCC*mec*IV  (USA1100) | 10±0.5 | 13±1.0 | 13±1.0 | 15±0.8 | 16±1.5 |
|  | ST5-SCC*mec*IV  (USA800) | 8±1.0 | 12±1.0 | 13±0.5 | 13±1.0 | 17±1.5 |

^*^VAN, vancomycin. VAN was used as control.

**References**

1. Biswas, R. *et al.* Activity of the major staphylococcal autolysin Atl. *FEMS Microbiol. Lett.* **259**, 260–268 (2006).

2. Ferreira FA *et al*. Impact of *agr* dysfunction on virulence profiles and infections associated with a novel methicillin-resistant *Staphylococcus aureus* (MRSA) variant of the lineage ST1-SCC*mec* IV. *BMC Microbiol*. **13**, 93. https://doi.org/ 10.1186/1471-2180-13-93 (2013).

3. Li, M. *et al.* Comparative analysis of virulence and toxin expression of global community-associated methicillin-resistant *Staphylococcus* *aureus* strains. *J. Infect. Dis.* **202**, 1866–1876 (2010).

4. Oliveira, D. C. & de Lencastre, H. Multiplex PCR strategy for rapid identification of structural types and variants of the *mec* element in methicillin-resistant *Staphylococcus* *aureus*. *Antimicrob. Agents Chemother.* **46**, 2155–2161 (2002).

5. Lauderdale, K. J., Boles, B. R., Cheung, A. L. & Horswill, A. R. Interconnections between Sigma B, *agr*, and proteolytic activity in *Staphylococcus* *aureus* biofilm maturation. *Infect. Immun.* **77**, 1623–1635 (2009).
